# Bioluminescence Imaging of Heme Oxygenase-1 Upregulation in the Gua Sha Procedure

**DOI:** 10.3791/1385

**Published:** 2009-08-28

**Authors:** Kenneth K. Kwong, Lenuta Kloetzer, Kelvin K. Wong, Jia-Qian Ren, Braden Kuo, Yan Jiang, Y. Iris Chen, Suk-Tak Chan, Geoffrey S. Young, Stephen T.C. Wong

**Affiliations:** Department of Radiology, Havard Medical School, Massachusetts General Hospital; Athinoula A. Martinos Center for Biomedical Imaging, Havard Medical School, Massachusetts General Hospital; Gastrointestinal Unit, Havard Medical School, Massachusetts General Hospital; Department of Medicine, Havard Medical School, Massachusetts General Hospital; Center for biotechnology and Informatics, The Methodist Hospital Research Institute; Department of Radiology, Weill Cornell Medical College of Cornell University; Bejing University of Chinese Medicine; Department of Health Technology and Informatics, The Hong Kong Polytechnic University - PolyU; Department of Radiology, Harvard Medical School

## Abstract

Gua Sha is a traditional Chinese folk therapy that employs skin scraping to cause subcutaneous microvascular blood extravasation and bruises. The protocol for bioluminescent optical imaging of HO-1-*luciferase *transgenic mice reported in this manuscript provides a rapid *in vivo* assay of the upregulation of the heme oxygenase-1 (HO-1) gene expression in response to the Gua Sha procedure. HO-1 has long been known to provide cytoprotection against oxidative stress. The upregulation of HO-1, assessed by the bioluminescence output, is thought to represent an antioxidative response to circulating hemoglobin products released by Gua Sha. Gua Sha was administered by repeated strokes of a smooth spoon edge over lubricated skin on the back or other targeted body part of the transgenic mouse until petechiae (splinter hemorrhages) or ecchymosis (bruises) indicative of extravasation of blood from subcutaneous capillaries was observed. After Gua Sha, bioluminescence imaging sessions were carried out daily for several days to follow the dynamics of HO-1 expression in multiple internal organs.

**Figure:**
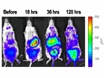


## Protocol

1. Female HO-1-*luciferase* transgenic mice (age of 4-6 weeks) can be purchased from Taconic Farms, Inc. Upon arrival*, *mice are placed in an animal housing facility for at least 4 days to allow accommodation.
2. Preparation for bioluminescence imaging:
  1. Weigh each HO-1 mouse. Animal weight is required for calculating the dosage of luciferin.
  2. Hair removal: Use a cotton swap to apply hair remover Nair® over the abdomen and/or back of the animal. Waiting for 5 to 10 seconds. Use a clean cotton swap to wipe off hair. Dip a piece of tissue in distilled water to wipe clean remaining hair.
  3. Place a non-fluorescent black paper (Strathmore Artagain® black paper) on the imaging platform of an IVIS 100 station to reduce background noise.
  4. Prepare luciferin (from Xenogen Corporation) solution at concentration of 7.5 mg/ml (dissolved in sterile H_2_O). The dose of luciferin is 65.5 mg/kg body weight. Thus, a 20 gram mouse requires intraperitoneal injection of 0.175 ml luciferin solution. To achieve this, a single injection volume of luciferin solution (0.175ml) is preloaded to a syringe with a 26-gauge needle
3. Gua Sha procedure: Gua Sha is applied only once before running the bioluminescence imaging protocol. Since Gua Sha is not painful, the mouse needs only to be briefly anesthetized by isoflurane to stay calm. Gua Sha procedure includes the following steps:
  1. Apply cooking oil or distilled water to lubricate skin areas targeted by Gua Sha a few times during the Gua Sha procedure.
  2. Repetitively scrape the hair-free region of the back of the mouse in gentle but firm force using a ceramic soup spoon or a plastic spoon.
  3. The scraping continues until the back skin turns red, which is a sign of subcutaneous blood extravasation, usually achieved within 2 to 3 minutes.
4. Bioluminescence imaging may be started immediately or several hours after Gua Sha as HO-1 upregulation is built up slowly over a few hours. The procedure for bioluminescence imaging is:
  1. Anesthetize the mouse in an anesthesia induction chamber filled with a mixture of isoflurane (1.5%) and medical grade air.
  2. Once the mouse is anesthetized, move the mouse to the imaging chamber on the IVIS 100 optical imaging station. Position the mouse in a supine position (abdomen up). The imaging chamber is continuously infused with 1.5% of isoflurane. The imaging platform is heated at 37°C to keep the mouse warm.
  3. Set the imaging acquisition at "medium binning" and set exposure time to be 30 seconds. Start to acquire images. Set the machine to repeat the imaging acquisition every three minutes, either manually or automatically. After the acquisition of the first image, a region of interest (ROI) is drawn to cover the abdominal, chest, and head area. This ROI is then copied and pasted to the following images using "Living Image®", software accompanying the IVIS 100 station.
  4. Signal intensity is measured in photons per second. Mark time for the signal intensity to reach its peak value and keep on imaging the mouse for about 5 to 10 minutes after the peak time.
  5. When the imaging acquisition at the supine position is done, flip the mouse over and lay it in the prone position (back up). Continue imaging the animal for 5 to 10 minutes with the ROI now drawn to cover the back and the head area in the use of the software "Living Image®". The choice of imaging the prone position after the supine position is arbitrary. One can choose to start with the prone position, depending on the primary organs of interest. Another option is to image the supine and prone positions at separate experiments.
  6. When the imaging acquisition at the prone position is finished, turn off isoflurane and move mouse from the imaging chamber to the induction chamber to recover. The induction chamber is now infused with medical air only and the mouse usually wakes up in less than one minute.
  7. Save imaging data for post-processing.
  8. After the initial Imaging study, additional imaging acquisitions are carried out for several days after Gua Sha. One sample schedule is repeated imaging at the 12^th^ hour, 24^th^ hour, 36^th^ hour, 48^th^ hour, 72^nd^ hour and 120^th^ hour but the exact timing of the imaging schedule is flexible. Additional imaging sessions may be added if a longer follow-up is needed. Before imaging, repeat the hair removing procedure if the hair has grown back.
5. For the control group, repeat all procedure but skip Gua Sha (step 3). If the same group of mice is used for both control and Gua Sha, the control experiment should be carried out preferably before the Gua sha procedure or at least one month afterwards.

### Representative Results:

The bioluminescence images in figure 1 show *in vivo* upregulation of HO-1 in response to Gua Sha. The graph in Figure 2 shows the quantitative temporal change over 120 hours in optical flux (photons/sec) from the whole body of the same mouse related to Gua Sha...


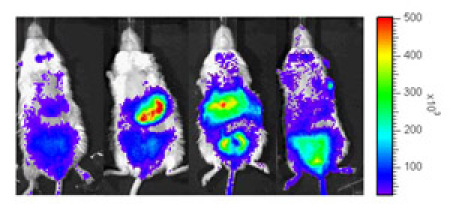
 **Figure 1.** From left to right, representative images of the front view (supine) of same mouse before Gua Sha, at 18 hours, 36 hours and 120 hours post Gua Sha, respectively.  After Gua Sha, one observes the progress of significant signal intensity increase in multiple organs which encompass regions of the gastrointestinal tract, the genital tract, the liver, kidneys (from the back view, not shown), and others. Please click here for a larger version of figure 1.


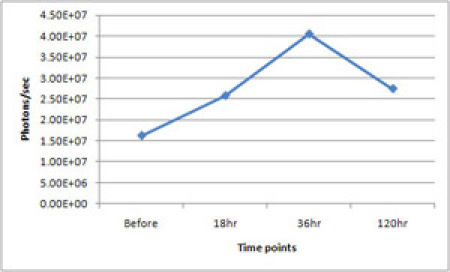
 **Figure 2.** Quantitative change of flux (photons/sec) from the whole body tracked over 120 hours following  Gua Sha in the same mouse of Figure 1. Please click here to see a larger version of figure 2.

## Discussion

Transcription of HO-1, an inducible form of heme oxygenase, is upregulated by many factors including heme, hydrogen peroxide, UV irradiation, hypoxia, and physical stresses.  Whole body imaging such as the reported HO-1 bioluminescence protocol provides a quick snapshot of systemic HO-1 expression in multiple organs. In small animals, bioluminescence imaging allows high-sensitivity in-vivo real-time quantitative longitudinal optical assessment of alterations in systemic gene expression, as shown.  Bioluminescence molecular imaging lowers the cost and increases the throughput of gene expression assays in small animals, making it practical to rapidly achieve the statistical power needed for investigation of complex systems biology hypothesis and to assess for systemic effects of proposed pharmaceutical and other therapeutic interventions.  One limitation is low spatial resolution of the anatomy. Newer optical imaging systems that have tomographic capability would alleviate the problem.  Image acquisition and registration by micro-CT or small animal MRI would help to properly identify the anatomy.  Other limitations are low light penetration through tissue and the need for transgenic animals. These essentially preclude translation to larger animals or human use, but are not a significant drawback for small animal systems biology or preclinical therapeutic hypothesis testing.

## References

[B0] Zhang W (2002). Selection of potential therapeutics based on in vivo spatiotemporal transcription patterns of heme oxygenase-1. J Mol Med.

[B1] Zhang W (2001). Rapid in vivo functional analysis of transgenes in mice using whole body imaging of luciferase expression. Transgenic Res.

[B2] Contag CH, Stevenson DK (2001). In vivo patterns of heme oxygenase-1 transcription. J Perinatol.

[B3] Nielsen A, Knoblauch NT, Dobos GJ, Michalsen A, Kaptchuk TJ (2007). The effect of Gua Sha treatment on the microcirculation of surface tissue: a pilot study in healthy subjects. Explore (NY).

[B4] Schwickert ME, Saha FJ, Braun M, Dobos GJ (2007). Gua Sha for migraine in inpatient withdrawal therapy of headache due to medication overuse. Forsch Komplementmed.

[B5] Tsai PS, Lee PH, Wang MY (2008). Demographics, training, and practice patterns of practitioners of folk medicine in Taiwan: a survey of the Taipei metropolitan area. J Altern Complement Med.

[B6] Nielsen A (2009). Gua sha research and the language of integrative medicine. J Bodyw Mov Ther.

[B7] Contag CH (1997). Visualizing gene expression in living mammals using a bioluminescent reporter. Photochem Photobiol.

[B8] Cui K, Xu X, Zhao H, Wong ST (2008). A quantitative study of factors affecting in vivo bioluminescence imaging. Luminescence.

[B9] Shibahara S, Yoshida T, Kikuchi G (1979). Mechanism of increase of heme oxygenase activity induced by hemin in cultured pig alveolar macrophages. Arch Biochem Biophys.

[B10] Lee PJ (1997). Hypoxia-inducible factor-1 mediates transcriptional activation of the heme oxygenase-1 gene in response to hypoxia. J Biol Chem.

[B11] Maines MD (1997). The heme oxygenase system: a regulator of second messenger gases. Annu Rev Pharmacol Toxicol.

[B12] Wunder C, Potter RF (2003). The heme oxygenase system: its role in liver inflammation. Curr Drug Targets Cardiovasc Haematol Disord.

[B13] Hoekstra KA, Godin DV, Cheng KM (2004). Protective role of heme oxygenase in the blood vessel wall during atherogenesis. Biochem Cell Biol.

[B14] Yang NC, Lu LH, Kao YH, Chau LY (2004). Heme oxygenase-1 attenuates interleukin-1beta-induced nitric oxide synthase expression in vascular smooth muscle cells. J Biomed Sci.

[B15] Morse D, Choi AM (2005). Heme oxygenase-1: from bench to bedside. Am J Respir Crit Care Med.

[B16] Ryter SW (2007). Protective functions of heme oxygenase-1 and carbon monoxide in the respiratory system. Antioxid Redox Signal.

[B17] Wilson K, Yu J, Lee A, Wu JC (2008). In vitro and in vivo bioluminescence reporter gene imaging of human embryonic stem cells. J Vis Exp.

[B18] Calabrese V (2009). Vitagenes, dietary antioxidants and neuroprotection in neurodegenerative diseases. Front Biosci.

[B19] Contag PR, Olomu IN, Stevenson DK, Contag CH (1998). Bioluminescent indicators in living mammals. Nat Med.

